# 2-(1-Ethyl-5-meth­oxy-1*H*-indol-3-yl)-*N*-isopropyl-2-oxoacetamide

**DOI:** 10.1107/S1600536811012815

**Published:** 2011-04-16

**Authors:** Xiao-Wei Tang, Tie-Liang Zhu, Hong Chen, Dan-Li Tian, Shao-Yu Shi

**Affiliations:** a School of Pharmacy, Tianjin Medical University, Tianjin 300070, People’s Republic of China; b Medical College of Chinese People’s Armed Police Forces, Tianjin 300162, People’s Republic of China; c Tianjin Key Laboratory for Biomarkers of Occupational and Environmental Hazards, Tianjin 300162, People’s Republic of China

## Abstract

In the title compound, C_16_H_20_N_2_O_3_, the crystal packing is stabilized by weak π–π stacking inter­actions [centroid–centroid distances = 3.577 (9) and 3.693 (9) Å] and inter­molecular C—H⋯O and N—H⋯O hydrogen-bond inter­actions. The C atoms of the *N*-isopropyl group are disordered over two sets of sites with occupancies of 0.61(3) and 0.39(3).

## Related literature

For the biological activity of the title compound and its derivatives, see: Souli *et al.* (2008 ▶); Chai *et al.* (2006 ▶); Radwan *et al.* (2007 ▶); Karthikeyan *et al.* (2009 ▶). For the preparation, see: Bacher *et al.* (2001 ▶). For bond lengths and angles in similar structures, see: Feng *et al.* (2008 ▶); Sonar *et al.* (2006 ▶).
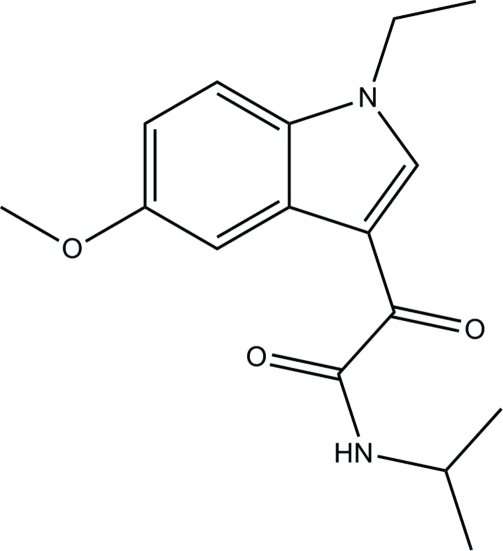


## Experimental

### 

#### Crystal data

C_16_H_20_N_2_O_3_
*M* *_r_* = 288.34
Orthorhombic, 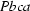

*a* = 11.593 (3) Å
*b* = 9.182 (2) Å
*c* = 27.796 (6) Å
*V* = 2958.8 (11) Å^3^
*Z* = 8
Mo *K*α radiation
μ = 0.09 mm^−1^
*T* = 113 K
0.30 × 0.24 × 0.10 mm

#### Data collection

Rigaku Saturn CCD area-detector diffractometer
Absorption correction: multi-scan (*CrystalClear*; Rigaku, 2005 ▶) *T*_min_ = 0.974, *T*_max_ = 0.991
25486 measured reflections
3527 independent reflections
3320 reflections with *I* > 2σ(*I*)
*R*_int_ = 0.053

#### Refinement

*R*[*F* ^2^ > 2σ(*F* ^2^)] = 0.047
*wR*(*F* ^2^) = 0.133
*S* = 1.12
3527 reflections
204 parameters
39 restraints
H atoms treated by a mixture of independent and constrained refinement
Δρ_max_ = 0.32 e Å^−3^
Δρ_min_ = −0.28 e Å^−3^

### 

Data collection: *CrystalClear* (Rigaku, 2005 ▶); cell refinement: *CrystalClear*; data reduction: *CrystalClear*; program(s) used to solve structure: *SHELXS97* (Sheldrick, 2008 ▶); program(s) used to refine structure: *SHELXL97* (Sheldrick, 2008 ▶); molecular graphics: *SHELXTL* (Sheldrick, 2008 ▶); software used to prepare material for publication: *SHELXTL*.

## Supplementary Material

Crystal structure: contains datablocks I, global. DOI: 10.1107/S1600536811012815/hg5020sup1.cif

Structure factors: contains datablocks I. DOI: 10.1107/S1600536811012815/hg5020Isup2.hkl

Additional supplementary materials:  crystallographic information; 3D view; checkCIF report

## Figures and Tables

**Table 1:** Hydrogen-bond geometry (Å, °)

*D*—H⋯*A*	*D*—H	H⋯*A*	*D*⋯*A*	*D*—H⋯*A*
C3—H3*A*⋯O2^i^	0.95	2.54	3.4874 (17)	172
N2—H2*B*⋯O3^ii^	0.894 (19)	2.149 (19)	2.9933 (17)	157.2 (16)

Symmetry codes: (i) 
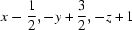
; (ii) 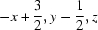.

**Fig. 1.**
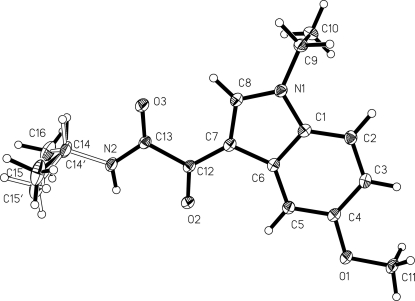
View of the title compound, with displacement ellipsoids drawn at the 40% probability level.

## supplementary crystallographic information

### Comment

Indole and their derivatives are well known as substances with biologically activity such as anti-cancer (Souli *et al.*, 2008), anti-virus (Chai *et al.*, 2006), anti-tubercular (Karthikeyan *et al.*, 2009), and anti-inflammatory (Radwan *et al.*, 2007). In recent years, our recent study is paying attention to synthesize different kinds of indole derivatives with improved bioactivities. In this paper, we reported the crystal structure of the title compound.

In title compound, C_16_H_20_N_2_O_3_, bond lengths and angles are normal and in good agreement with those reported previously (Feng *et al.*, 2008; Sonar, *et al.*, 2006). π—π interactions are indicated by the short distance (*Cg*1···*Cg*2 distance of 3.693 (9) Å, *Cg*2···*Cg*2 distance of 3.577 (9)Å symmetry code: 1 - *x*,1 - *y*,-*z*) between the centroids of the pyrrole ring (N1/C1/C6—C8) (*Cg*1) and benzene ring C1—C6 (*Cg*2) (Table 1). There are weaker C—H···O and N—H···O intermolecular interactions, which stabilized the structure (Table 1).

### Experimental

The target compound was synthesized by three steps. Under ice bath, DMF solution of 5-methoxy indole was added dropwise to DMF solution of NaH 0.5 h later, ethyl bromide was added. The reaction solvent add into ice water, and the compound is separated from the liquid after stirring. Oxalyl chloride was added dropwise to the 1-ethyl-methoxy indole in dry ether, 1-ethyl-methoxy indole-3-yl-glyoxyl chloride which was the crude product, propan-2-amine, two drops of triethylamine were in dry dichloromethane. The reaction mixture was washed with water and dried over Na_2_SO_4_ and concentrated in vacuo (Bacher *et al.*, 2001). The residue was resolved in a methanol solution. Slow evaporatin over two week at room temperature gave light-yellow crystals suitable for X-ray analysis.

### Refinement

All C H atoms were found on difference maps, with C—H = 0.95–1.00 Å and H atoms bonded N were refined freely with N—H = 0.89 Å and included in the final cycles of refinement using a riding model, with *U*_iso_(H) = 1.2*U*_eq_(C) for aryl and methylene H atoms and 1.5*U*_eq_(C) for the methyl H atoms.

### Crystal data

**Table:**

C_16_H_20_N_2_O_3_	*F*(000) = 1232
*M**_r_* = 288.34	*D*_x_ = 1.295 Mg m^−^^3^
Orthorhombic, *P**b**c**a*	Mo *K*α radiation, λ = 0.71073 Å
Hall symbol: -P 2ac 2ab	Cell parameters from 9914 reflections
*a* = 11.593 (3) Å	θ = 1.9–27.9°
*b* = 9.182 (2) Å	µ = 0.09 mm^−^^1^
*c* = 27.796 (6) Å	*T* = 113 K
*V* = 2958.8 (11) Å^3^	Prism, colorless
*Z* = 8	0.30 × 0.24 × 0.10 mm

### Data collection

**Table:**

Rigaku Saturn CCD area-detector diffractometer	3527 independent reflections
Radiation source: rotating anode	3320 reflections with *I* > 2σ(*I*)
multilayer	*R*_int_ = 0.053
Detector resolution: 14.63 pixels mm^-1^	θ_max_ = 27.9°, θ_min_ = 1.5°
ω and φ scans	*h* = −14→15
Absorption correction: multi-scan (*CrystalClear*; Rigaku, 2005)	*k* = −12→9
*T*_min_ = 0.974, *T*_max_ = 0.991	*l* = −36→36
25486 measured reflections	

### Refinement

**Table:**

Refinement on *F*^2^	Primary atom site location: structure-invariant direct methods
Least-squares matrix: full	Secondary atom site location: difference Fourier map
*R*[*F*^2^ > 2σ(*F*^2^)] = 0.047	Hydrogen site location: inferred from neighbouring sites
*wR*(*F*^2^) = 0.133	H atoms treated by a mixture of independent and constrained refinement
*S* = 1.12	*w* = 1/[σ^2^(*F*_o_^2^) + (0.0661*P*)^2^ + 0.8297*P*] where *P* = (*F*_o_^2^ + 2*F*_c_^2^)/3
3527 reflections	(Δ/σ)_max_ = 0.001
204 parameters	Δρ_max_ = 0.32 e Å^−^^3^
39 restraints	Δρ_min_ = −0.28 e Å^−^^3^

### Special details

**Table:**

Geometry. All e.s.d.'s (except the e.s.d. in the dihedral angle between two l.s. planes) are estimated using the full covariance matrix. The cell e.s.d.'s are taken into account individually in the estimation of e.s.d.'s in distances, angles and torsion angles; correlations between e.s.d.'s in cell parameters are only used when they are defined by crystal symmetry. An approximate (isotropic) treatment of cell e.s.d.'s is used for estimating e.s.d.'s involving l.s. planes.
Refinement. Refinement of *F*^2^ against ALL reflections. The weighted *R*-factor *wR* and goodness of fit *S* are based on *F*^2^, conventional *R*-factors *R* are based on *F*, with *F* set to zero for negative *F*^2^. The threshold expression of *F*^2^ > σ(*F*^2^) is used only for calculating *R*-factors(gt) *etc*. and is not relevant to the choice of reflections for refinement. *R*-factors based on *F*^2^ are statistically about twice as large as those based on *F*, and *R*- factors based on ALL data will be even larger.

### Fractional atomic coordinates and isotropic or equivalent isotropic displacement parameters (Å^2^)

**Table:**

	*x*	*y*	*z*	*U*_iso_*/*U*_eq_	Occ. (<1)
O1	0.47198 (9)	0.63365 (11)	0.45512 (3)	0.0265 (2)	
O2	0.65875 (8)	0.73051 (10)	0.62010 (3)	0.0242 (2)	
O3	0.64406 (8)	1.05760 (10)	0.68489 (3)	0.0233 (2)	
N1	0.37396 (10)	1.06105 (12)	0.58694 (4)	0.0201 (2)	
N2	0.75248 (11)	0.85420 (14)	0.69650 (4)	0.0247 (3)	
C1	0.38287 (11)	0.95977 (14)	0.54960 (4)	0.0188 (3)	
C2	0.31491 (12)	0.94441 (14)	0.50881 (4)	0.0208 (3)	
H2A	0.2511	1.0070	0.5031	0.025*	
C3	0.34338 (11)	0.83450 (15)	0.47660 (5)	0.0210 (3)	
H3A	0.2987	0.8211	0.4483	0.025*	
C4	0.43820 (11)	0.74299 (14)	0.48577 (4)	0.0200 (3)	
C5	0.50502 (11)	0.75732 (14)	0.52708 (4)	0.0198 (3)	
H5A	0.5681	0.6938	0.5330	0.024*	
C6	0.47675 (11)	0.86775 (14)	0.55956 (4)	0.0177 (3)	
C7	0.52557 (11)	0.91743 (14)	0.60485 (4)	0.0185 (3)	
C8	0.45861 (11)	1.03582 (14)	0.61905 (4)	0.0191 (3)	
H8A	0.4710	1.0911	0.6475	0.023*	
C9	0.27776 (12)	1.16154 (15)	0.59397 (5)	0.0244 (3)	
H9A	0.2564	1.2055	0.5627	0.029*	
H9B	0.3020	1.2411	0.6158	0.029*	
C10	0.17353 (13)	1.08439 (16)	0.61518 (5)	0.0284 (3)	
H10A	0.1105	1.1544	0.6194	0.043*	
H10B	0.1941	1.0425	0.6464	0.043*	
H10C	0.1487	1.0066	0.5934	0.043*	
C11	0.40229 (13)	0.60947 (16)	0.41358 (5)	0.0261 (3)	
H11A	0.4349	0.5295	0.3946	0.039*	
H11B	0.4003	0.6982	0.3940	0.039*	
H11C	0.3238	0.5841	0.4236	0.039*	
C12	0.61865 (11)	0.85066 (14)	0.63061 (4)	0.0180 (3)	
C13	0.67303 (11)	0.93253 (14)	0.67372 (4)	0.0189 (3)	
C15	0.9362 (6)	0.9639 (14)	0.7211 (3)	0.0447 (16)	0.61 (3)
H15A	0.9270	1.0323	0.6943	0.067*	0.61 (3)
H15B	0.9831	0.8809	0.7106	0.067*	0.61 (3)
H15C	0.9746	1.0132	0.7479	0.067*	0.61 (3)
C14	0.81996 (14)	0.91109 (16)	0.73695 (5)	0.0291 (3)	0.61 (3)
H14A	0.7777	0.9993	0.7485	0.035*	0.61 (3)
C15'	0.9489 (8)	0.911 (2)	0.7214 (5)	0.048 (3)	0.39 (3)
H15D	0.9596	0.9779	0.6943	0.072*	0.39 (3)
H15E	0.9712	0.8122	0.7115	0.072*	0.39 (3)
H15F	0.9971	0.9418	0.7485	0.072*	0.39 (3)
C14'	0.81996 (14)	0.91109 (16)	0.73695 (5)	0.0291 (3)	0.39 (3)
H14B	0.7935	1.0102	0.7470	0.035*	0.39 (3)
C16	0.81764 (17)	0.8043 (2)	0.77817 (5)	0.0408 (4)	
H16A	0.7405	0.7968	0.7907	0.049*	
H16B	0.8425	0.7108	0.7667	0.049*	
H16C	0.8685	0.8368	0.8027	0.049*	
H2B	0.7703 (15)	0.765 (2)	0.6858 (6)	0.031 (5)*	

### Atomic displacement parameters (Å^2^)

**Table:**

	*U*^11^	*U*^22^	*U*^33^	*U*^12^	*U*^13^	*U*^23^
O1	0.0291 (5)	0.0292 (5)	0.0212 (5)	0.0041 (4)	−0.0062 (4)	−0.0094 (4)
O2	0.0260 (5)	0.0223 (5)	0.0243 (5)	0.0028 (4)	−0.0032 (4)	−0.0065 (4)
O3	0.0265 (5)	0.0199 (5)	0.0234 (5)	−0.0003 (4)	−0.0028 (4)	−0.0050 (4)
N1	0.0251 (6)	0.0172 (5)	0.0181 (5)	0.0007 (4)	−0.0016 (4)	−0.0008 (4)
N2	0.0303 (6)	0.0212 (6)	0.0227 (5)	0.0025 (5)	−0.0089 (5)	−0.0065 (5)
C1	0.0229 (6)	0.0165 (6)	0.0171 (6)	−0.0022 (5)	0.0016 (5)	0.0011 (5)
C2	0.0235 (7)	0.0206 (6)	0.0183 (6)	0.0003 (5)	−0.0015 (5)	0.0026 (5)
C3	0.0232 (7)	0.0220 (6)	0.0178 (6)	−0.0030 (5)	−0.0025 (5)	0.0010 (5)
C4	0.0228 (6)	0.0196 (6)	0.0176 (6)	−0.0027 (5)	0.0007 (5)	−0.0018 (5)
C5	0.0199 (6)	0.0206 (6)	0.0189 (6)	−0.0011 (5)	−0.0002 (5)	−0.0005 (5)
C6	0.0188 (6)	0.0181 (6)	0.0162 (5)	−0.0046 (5)	0.0009 (4)	0.0006 (5)
C7	0.0208 (6)	0.0188 (6)	0.0157 (5)	−0.0042 (5)	0.0014 (4)	−0.0003 (5)
C8	0.0226 (6)	0.0181 (6)	0.0165 (6)	−0.0031 (5)	0.0000 (5)	0.0002 (5)
C9	0.0318 (7)	0.0185 (6)	0.0228 (6)	0.0059 (5)	−0.0032 (5)	−0.0005 (5)
C10	0.0274 (7)	0.0273 (7)	0.0304 (7)	0.0082 (6)	−0.0016 (6)	−0.0013 (6)
C11	0.0308 (7)	0.0286 (7)	0.0189 (6)	−0.0015 (6)	−0.0052 (5)	−0.0062 (5)
C12	0.0189 (6)	0.0197 (6)	0.0154 (5)	−0.0038 (5)	0.0024 (4)	−0.0011 (4)
C13	0.0204 (6)	0.0208 (6)	0.0154 (5)	−0.0035 (5)	0.0015 (5)	−0.0013 (4)
C15	0.053 (2)	0.049 (4)	0.0325 (17)	−0.027 (2)	−0.0132 (15)	0.002 (2)
C14	0.0383 (8)	0.0248 (7)	0.0240 (7)	0.0002 (6)	−0.0138 (6)	−0.0066 (5)
C15'	0.051 (4)	0.059 (6)	0.035 (3)	−0.032 (4)	−0.009 (3)	−0.002 (4)
C14'	0.0383 (8)	0.0248 (7)	0.0240 (7)	0.0002 (6)	−0.0138 (6)	−0.0066 (5)
C16	0.0567 (11)	0.0436 (10)	0.0222 (7)	−0.0091 (8)	−0.0086 (7)	−0.0035 (6)

### Geometric parameters (Å, °)

**Table:**

O1—C4	1.3739 (15)	C9—C10	1.520 (2)
O1—C11	1.4264 (16)	C9—H9A	0.9900
O2—C12	1.2323 (16)	C9—H9B	0.9900
O3—C13	1.2361 (16)	C10—H10A	0.9800
N1—C8	1.3465 (16)	C10—H10B	0.9800
N1—C1	1.3974 (16)	C10—H10C	0.9800
N1—C9	1.4606 (17)	C11—H11A	0.9800
N2—C13	1.3291 (18)	C11—H11B	0.9800
N2—C14	1.4659 (17)	C11—H11C	0.9800
N2—H2B	0.894 (19)	C12—C13	1.5487 (17)
C1—C2	1.3879 (18)	C15—C14	1.499 (5)
C1—C6	1.4053 (18)	C15—H15A	0.9800
C2—C3	1.3888 (18)	C15—H15B	0.9800
C2—H2A	0.9500	C15—H15C	0.9800
C3—C4	1.4069 (19)	C14—C16	1.508 (2)
C3—H3A	0.9500	C14—H14A	1.0000
C4—C5	1.3913 (18)	C15'—H15D	0.9800
C5—C6	1.3966 (17)	C15'—H15E	0.9800
C5—H5A	0.9500	C15'—H15F	0.9800
C6—C7	1.4537 (17)	C16—H16A	0.9632
C7—C8	1.3928 (18)	C16—H16B	0.9595
C7—C12	1.4329 (18)	C16—H16C	0.9502
C8—H8A	0.9500		
			
C4—O1—C11	117.02 (11)	C9—C10—H10B	109.5
C8—N1—C1	108.90 (11)	H10A—C10—H10B	109.5
C8—N1—C9	125.21 (11)	C9—C10—H10C	109.5
C1—N1—C9	125.18 (11)	H10A—C10—H10C	109.5
C13—N2—C14	122.84 (12)	H10B—C10—H10C	109.5
C13—N2—H2B	119.8 (12)	O1—C11—H11A	109.5
C14—N2—H2B	117.2 (12)	O1—C11—H11B	109.5
C2—C1—N1	129.23 (12)	H11A—C11—H11B	109.5
C2—C1—C6	122.64 (12)	O1—C11—H11C	109.5
N1—C1—C6	108.12 (11)	H11A—C11—H11C	109.5
C1—C2—C3	117.75 (12)	H11B—C11—H11C	109.5
C1—C2—H2A	121.1	O2—C12—C7	123.27 (12)
C3—C2—H2A	121.1	O2—C12—C13	117.68 (11)
C2—C3—C4	120.19 (12)	C7—C12—C13	119.05 (11)
C2—C3—H3A	119.9	O3—C13—N2	124.85 (12)
C4—C3—H3A	119.9	O3—C13—C12	122.32 (12)
O1—C4—C5	114.98 (12)	N2—C13—C12	112.83 (11)
O1—C4—C3	123.14 (11)	C14—C15—H15A	109.5
C5—C4—C3	121.88 (12)	C14—C15—H15B	109.5
C4—C5—C6	118.12 (12)	H15A—C15—H15B	109.5
C4—C5—H5A	120.9	C14—C15—H15C	109.5
C6—C5—H5A	120.9	H15A—C15—H15C	109.5
C5—C6—C1	119.40 (12)	H15B—C15—H15C	109.5
C5—C6—C7	134.12 (12)	N2—C14—C15	111.7 (3)
C1—C6—C7	106.47 (11)	N2—C14—C16	109.97 (12)
C8—C7—C12	127.74 (11)	C15—C14—C16	116.8 (4)
C8—C7—C6	105.86 (11)	N2—C14—H14A	105.9
C12—C7—C6	126.28 (12)	C15—C14—H14A	105.9
N1—C8—C7	110.65 (11)	C16—C14—H14A	105.9
N1—C8—H8A	124.7	H15D—C15'—H15E	109.5
C7—C8—H8A	124.7	H15D—C15'—H15F	109.5
N1—C9—C10	111.38 (11)	H15E—C15'—H15F	109.5
N1—C9—H9A	109.4	C14—C16—H16A	109.8
C10—C9—H9A	109.4	C14—C16—H16B	109.0
N1—C9—H9B	109.4	H16A—C16—H16B	109.6
C10—C9—H9B	109.4	C14—C16—H16C	109.3
H9A—C9—H9B	108.0	H16A—C16—H16C	109.7
C9—C10—H10A	109.5	H16B—C16—H16C	109.5
			
C8—N1—C1—C2	−179.77 (13)	C5—C6—C7—C12	−5.2 (2)
C9—N1—C1—C2	9.5 (2)	C1—C6—C7—C12	175.99 (12)
C8—N1—C1—C6	0.47 (14)	C1—N1—C8—C7	−0.64 (15)
C9—N1—C1—C6	−170.26 (12)	C9—N1—C8—C7	170.09 (12)
N1—C1—C2—C3	179.21 (12)	C12—C7—C8—N1	−175.62 (12)
C6—C1—C2—C3	−1.05 (19)	C6—C7—C8—N1	0.54 (14)
C1—C2—C3—C4	0.07 (19)	C8—N1—C9—C10	−92.41 (15)
C11—O1—C4—C5	176.62 (12)	C1—N1—C9—C10	76.84 (16)
C11—O1—C4—C3	−3.38 (19)	C8—C7—C12—O2	165.58 (13)
C2—C3—C4—O1	−179.06 (12)	C6—C7—C12—O2	−9.8 (2)
C2—C3—C4—C5	0.9 (2)	C8—C7—C12—C13	−14.81 (19)
O1—C4—C5—C6	179.04 (11)	C6—C7—C12—C13	169.78 (11)
C3—C4—C5—C6	−0.96 (19)	C14—N2—C13—O3	−3.0 (2)
C4—C5—C6—C1	−0.01 (18)	C14—N2—C13—C12	176.90 (12)
C4—C5—C6—C7	−178.74 (13)	O2—C12—C13—O3	174.90 (12)
C2—C1—C6—C5	1.04 (19)	C7—C12—C13—O3	−4.74 (18)
N1—C1—C6—C5	−179.18 (11)	O2—C12—C13—N2	−4.97 (17)
C2—C1—C6—C7	−179.92 (12)	C7—C12—C13—N2	175.39 (12)
N1—C1—C6—C7	−0.13 (14)	C13—N2—C14—C15	−97.9 (6)
C5—C6—C7—C8	178.61 (14)	C13—N2—C14—C16	130.80 (15)
C1—C6—C7—C8	−0.23 (13)		

### Hydrogen-bond geometry (Å, °)

**Table:**

*D*—H···*A*	*D*—H	H···*A*	*D*···*A*	*D*—H···*A*
C3—H3A···O2^i^	0.95	2.54	3.4874 (17)	172
N2—H2B···O3^ii^	0.894 (19)	2.149 (19)	2.9933 (17)	157.2 (16)
Cg1—···.Cg2^iii^	.	.	3.693 (9)	.
Cg2—···.Cg2^iii^	.	.	3.577 (9)	.

Symmetry codes: (i) *x*−1/2, −*y*+3/2, −*z*+1; (ii) −*x*+3/2, *y*−1/2, *z*; (iii) −*x*+1, −*y*+1, −*z*.
